# Optimized Biotransformation of Icariin into Icariside II by *β*-Glucosidase from* Trichoderma viride* Using Central Composite Design Method

**DOI:** 10.1155/2016/5936947

**Published:** 2016-02-14

**Authors:** Tao Cheng, Jun Yang, Tong Zhang, Yi-Shun Yang, Yue Ding

**Affiliations:** ^1^Experiment Center of Teaching & Learning, Shanghai University of Traditional Chinese Medicine, Shanghai 201203, China; ^2^Shanghai Xiangshan Hospital of Traditional Chinese Medicine, No. 528 in Middle of Renaissance Road, Shanghai 201203, China; ^3^Department of Pharmaceutics, University of Washington, Seattle, WA 98105, USA

## Abstract

A crude *β*-glucosidase has been produced from* Trichoderma viride* and used to explore a simple method to prepare icariside II from icariin. The crude enzyme has been studied by zymography method and used for hydrolysis of ICA. To achieve a high conversion rate of ICA, various factors have been studied including pH, reaction time, temperature, initial concentration of enzyme, and initial concentration of ICA through central composite design experiments. In the condition of the optimum hydrolysis parameters with pH 4.0, 41°C, 1.0 mg/mL ICA, and 9.8 U/mL crude *β*-glucosidase, the conversion rate of ICA reached 95.03% at 1 h. Moreover, the cytotoxicity test showed that ICA II performed inhibition effects on proliferation of A549 cell, while ICA has no cytotoxicity. It indicated that the hydrolysis transformation study of ICA is valuable for exploration of active new drugs.

## 1. Introduction

Epimedii folium, also named yin yang huo, is dried leaves from* Epimedium brevicornum* Maxim,* Epimedium sagittatum* (Sieb. et Zucc.) Maxim, and* Epimedium koreanum* Nakai, which has been used as a tonic herb prescribed to “nourish the kidney and strengthen the bone” for thousands of years [1]. The modern pharmacology researches indicated that Epimedii folium can be used to treat hyperglycaemia, diabetic complication, reproductive dysfunction disease, and so on [2–6]. It is popular to prepare medicated wine or health protection tea in Asia. In addition, a large amount of Epimedii folium has been exported to Japan and America each year for people's healthy industry, which are worthy of millions of dollars [7].

**Figure 1:**
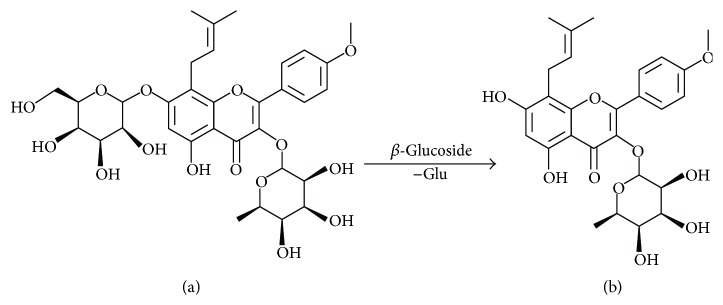
The structures of icariin and icariside II (a) ICA; (b) ICA II.

Icariin (ICA), a natural flavonoid glycoside, is supposed to be the major constituent with the content above 0.5% in herba Epimedii [8]. ICA has been shown to possess anti-inflammatory, antidepressant, antioxidative, antiatherosclerosis, anticancer, and insulin resistance activity [9–13]. Icariside II (ICA II, Baohuoside I) is one kind of metabolites of ICA, which is a loss of the glycosyl moiety at the C-7 position of ICA (Figure 1) [14]. Metabolism and pharmacokinetics studies have revealed that ICA is metabolized by intestinal flora in vivo and absorbed in form of ICA II [15–18]. Though both ICA and ICA II exhibit many common pharmacological effects [19–21], ICA II shows a stronger biological activity in some pharmacological effects compared with ICA, such as improving endothelial cells [19], promoting the osteogenic differentiation of bone marrow mesenchymal stem cells [20], and extending the life of* C. elegans* [21]. Cai et al.'s research demonstrated that ICA II treatment induces a similar extension with relatively lower dosage compared with ICA, and the lifespan extension effect of ICA II is dependent on the insulin/IGF-1 signaling (IIS) [21]. Therefore, applying ICA II directly may have better clinical significance, and it is worthy to explore a stable process to prepare a large amount of ICA II for its further pharmaceutical research.

Chromatography technology was the traditional and original method for preparing ICA II, which isolated and purified it from herbs directly [22], but it can offer you only small amount of ICA II as the content of natural ICA II in herba Epimedii was about only 0.01% g/g, which is about 1/60–1/6 that of ICA. However, transformation of ICA into ICA II can offer more ICA II by chemical or enzymatic hydrolysis. Comparing with the chemical hydrolysis process, biotransformation method using specific enzyme is efficient and effective, which has advantages of high conversion rate of ICA, mild reaction conditions, and less byproducts. According to the molecular structure of ICA, *β*-glucosidase would be the specific catalyst in the process of biotransformation of ICA.* Trichoderma* is one of the most efficient cellulases producer organisms, which can be used for the production of *β*-glucosidase [23, 24]. In the present work, a crude *β*-glucosidase has been produced from* Trichoderma viride* and used to explore a simple and feasible method to prepare ICA II from ICA. The crude enzyme has been studied by zymography method and *β*-glucosidase is identified as an efficient catalyst.

Central composite design (CCD) is a statistical technique and widely used to model complex process [25, 26], which requires a few of experiments comparing with conventional methods. In this study, CCD method was adopted to optimize the parameters of biotransformation of ICA by investigating three key elements: the initial concentration of substrate and pH coupled with temperature. To the best of our knowledge, this process costs less time to gain higher (>90%) hydrolysis rate than other researches.

## 2. Materials and Methods

### 2.1. Materials and Reagents

ICA, ICA II, and 4-methylumbelliferyl-*β*-*D*-glucopyranoside were procured from Shanghai Yuanye Bio-Technology Co., Ltd. Sodium acetate, acetic acid, sodium carbonate, ethyl acetate, and formic acid were purchased from Sinopharm Chemical Reagent Co., Ltd. (Shanghai, China). Rapid Silver Staining Kit was acquired from Beyotime Institute of Biotechnology (Haimen, China). Methanol used in chromatography was of HPLC grade, from Merck (Darmstadt, Germany).

### 2.2. Enzyme Preparation

The homogeneous inoculum of* Trichoderma viride* AS 3.3711 was developed in a flask with potato dextrose broth at 30 ± 1°C for 5 days. The fungal strain* Trichoderma viride* was cultured in the solid state medium of orange peel (50% w/w moisture). The initial pH value of the medium was adjusted to 5. The autoclaved medium was inoculated with 5 mL of freshly prepared fungal inoculum and incubated at 30 ± 1°C. Extracted *β*-glucosidase from the fermented biomass by adding citrate buffer (0.05 M of pH 4.8) in 1 : 10 ratio and the flasks were shaken at 120 rpm for 30 min. The contents were filtered with muslin cloth and filtrates were centrifuged at 10,000 g for 10 min. The supernatants were carefully collected and freeze-dried. The freeze-dried powders as crude *β*-glucosidase were used for hydrolysis process study of ICA.

### 2.3. Zymogram Analysis

The above crude *β*-glucosidase was dissolved in sodium acetate buffer (pH 5.0, 20 mM), separated, and retained by Native-PAGE (Mini-PROTEAN Tetra Electrophoresis, Bio-Rad, USA). Then, the zymogram analysis was performed following the procedure in previous paper [27]. After electrophoresis, one gel was immersed in sodium acetate buffer (pH 5.0, 20 mM) containing 10 mM* p*NPG and incubated for 30 min at 50°C. Then gel was immersed in 1 M sodium carbonate to stop the reaction. The other gel was incubated in 20 mM sodium acetate buffer (pH 5.0) containing 10 mg/mL MUG (pH 5.0) for 30 min at 50°C and was observed under UV light (366 nm). The target bands in Native-PAGE gels were cut down referring to above chromogenic reaction, mashed, and soaked in 20 mM sodium acetate buffer (pH 5.0) at −20°C. After 2 h they were centrifuged in 8000 g/min at 4°C for 1 min by high speed centrifuge Eppendorf (5810R, Germany). The supernate was analyzed by SDS-PAGE and stained by silver staining method.

### 2.4. Determination of ICA II by High-Performance Liquid Chromatography (HPLC)

The analysis of ICA II was performed by HPLC system equipped with a UV detector at 270 nm and a Diamonsil C_18_ column (250 × 4.6 mm, 5 *μ*m; Dikma Technologies Co., Ltd.) was used under 30°C on an Agilent 1200 instrument. The mobile phases consisted of methanol (A) and water (B) with ratio of 78 : 22, at a flow rate of 1.0 mL/min. The selectivity of the method was investigated by analyzing blank hydrolysis liquid (no ICA and ICA II in it), a standard working solution sample consisting of ICA and ICA II, and a real sample after hydrolysis of ICA. The linear relationship of the method was evaluated by establishing the calibration curves by plotting peak area ratios of ICA II versus the concentrations of ICA II. The calibration curves were established at seven different concentrations of 4.94, 9.88, 24.7, 49.4, 98.8, 247, and 494 *μ*g/mL for ICA II. All the standard solutions were prepared by diluting a stock solution of ICA II (988 *μ*g/mL) in methanol. Each point of the calibration curve was injected in triplicate. The LLOQ was defined as the concentration giving a signal-to-noise ratio of at least 10. The intraday precision study was done by injecting the prepared standard solution at three concentration levels (9.88, 98.8, and 494 *μ*g/mL, six replicates for each concentration level) in one day. Interday studies were done by injecting the standards samples (9.88, 98.8, and 494 *μ*g/mL, triplicate for each concentration level) over three consecutive days. The precision of the method at each concentration level was expressed as the relative standard deviation (RSD). The accuracy was assessed through recovery tests, which was done by the standard addition method. The samples were prepared by adding a certain amount of ICA II standard solution into the samples whose content of ICA II has also been determined. The added amounts of ICA II have been set at three levels which were 80%, 100%, and 120% of the amount of ICA II in the samples. The accuracy was expressed as recovery rates and RSD. The suitability of the precision and accuracy was assessed by the following criteria: the RSDs were less than 3%, and recovery rates were between 95% and 105%. Sample stability was carried out by leaving spiked sample solutions in tightly capped volumetric flasks at room temperature for 12 h after preparation. Contents of ICA II were determined at 0 h, 2 h, 4 h, 8 h, and 12 h and its stability was expressed as RSD, which should be less than 3%.

### 2.5. Monofactor Experiments of ICA Biotransformation into ICA II

The hydrolysis reaction was carried out by the following procedure: 11 mL buffer solution of ICA was transferred to a 50 mL erlenmeyer flask together with 1 mL *β*-glucosidase buffer solution. After vibration with constant temperature shaker incubator at 200 rpm/min for a period of time, the reaction liquid was extracted by 3 volumes of acetic ether. The collection was evaporated to dryness at 60°C. The residues were dissolved in methanol and filtered with 0.45 *μ*m microporous membrane that was analyzed by HPLC system. To obtain higher rate of enzymatic hydrolysis, the hydrolysis conditions including PH, temperature, time, and the amount of enzyme (U/mL) and concentration of substrate (mg/mL) have been optimized by monofactor experiments. To facilitate the research, *β*-glucosidase was dissolved in buffer solution at concentration of 19.6 U/mL. The hydrolysis rate of ICA could be calculated according to the following equation:

(1) The  hydrolysis  rate  of  ICA  %=Total  of  C  ICA  II×676.65Total  of  C  ICA×514.13×100%.

In order to optimize the amount of *β*-glucosidase in the process of hydrolysis, the other factors including pH of buffer solution, incubation temperature, incubation time, and initial concentration of ICA were fixed at 5.0, 40°C, 1 hour, and 1 mg/mL. The enzyme amount was varied with the volume of the enzyme (0.1, 0.2, 0.5, 1, and 2 mL) at the same concentration (19.6 U/mL). For optimization of the incubation times (30, 60, 90, 120, and 180 min) for enzymatic hydrolysis, pH of buffer solution, incubation temperature, amount of enzyme, and initial concentration of ICA were fixed at conditions including 5.0, 40°C, 1 mL, and 1 mg/mL. To optimize the incubation temperature (40, 45, 50, 55, and 60°C) for the reaction, the pH of buffer solution, the amount of enzyme, the incubation time, and the initial concentration of ICA were fixed at conditions of 5.0, 1 mL, 1 hour, and 1 mg/mL, in sequence. pH (3.5, 4.0, 5.0, 6.0, 7.0, and 8.0) of buffer solution will be optimized by fixing other factors that the enzyme amount, the incubation temperature, the incubation time, and the initial concentration of ICA were fixed at 1 mL, 40°C, 1 hour, and 1 mg/mL. Finally, the initial concentration of ICA (0.1, 0.3, 0.5, 0.8, 1, and 2 mg/mL) will be optimized when other factors including pH of buffer solution, incubation temperature, incubation time, and amount of *β*-glucosidase were fixed at 5.0, 40°C, 1 hour, and 1 mL. The hydrolysis rate of ICA will be taken as an index in all the above experiments.

### 2.6. Optimization of ICA Biotransformation into ICA II with Central Composite Design (CCD)

After evaluating the effects of each factor on hydrolysis rate of ICA by monofactor method, CCD was introduced to optimize the three important parameters: pH (*X*_1_), temperature (*X*_2_), and concentration of substrate (*X*_3_), to achieve the optimum hydrolysis efficiency. Each independent variable was studied at five different levels as per CCD in three variables with a total of 20 experiments. The range of variable pH, temperature, and concentration of substrate was chosen according to above monofactor experiments. The plan of CCD in coded levels of the three independent variables is shown in Table 1.

The three variables were coded to the following equation:

(2) $$\begin{array}{c} X_{i}=\frac{\left(X_{i}-X_{0}\right)}{\delta X_{i}}, \end{array}$$

where *X*_*i*_ is the dimensionless value of an independent variable, *X*_*i*_ is the real value of a variable, *X*_0_ is the midpoint of *X*_*i*_; and *δX*_*i*_ is the step change in *X*_*i*_, *i* = 1,2, 3.

Conversion rate of ICA (response) was explained as a second-order response surface model in three independent variables:

(3) $$\begin{array}{c} \hat{Y_{ı}}=\beta_{0}+\overset{3}{\underset{i=1}{\sum }}\beta_{i}X_{i}+\overset{3}{\underset{i=1}{\sum }}\beta_{ij}X_{i}^{2}+\overset{3}{\underset{i,j=1}{\sum }}\beta_{ij}X_{i}X_{j}, \end{array}$$

where *β*_0_, *β*_*i*_, *β*_*II*_, and *β*_*ij*_ represent, respectively, the constant process effect in total, the linear, quadratic effect of *X*_*i*_, and the interaction effect between *X*_*i*_ and *X*_*j*_ on conversion of ICA. The results were analyzed by using the Design Expert 7.0.0 Trial (State Ease Inc., Minneapolis, MN, USA).

### 2.7. The Purification and Identification of the Product

The hydrolysates were dissolved in methanol at 70°C with the concentration of 100 mg/mL and crystallized in room temperature. The product was shown in form of yellow crystalline powder, which was identified by IR, UV, ^1^H-NMR, and ^13^C-NMR.

### 2.8. Cytotoxicity of ICA and ICA II on Human Lung Adenoma A549 Cells

To evaluate the efficacy of ICA and ICA II, the human lung adenoma A549 cells were chosen in cell test. The cells were cultured in Dulbecco's modified Eagle's medium supplemented with 10% fetal bovine serum and 1% PSN antibiotic (DMEM solution) at 37°C, 5% CO_2_. The viability of A549 cells was assessed by the MTT (3-(4,5-dimethylthiazol-2-yl)-2,5-diphenyltetrazolium bromide) assay after exposure to either ICA or ICA II. Cells were seeded in 96-well plates at a density of 10^5^ cells/mL and were incubated with ICA and ICA II at different concentrations (0.0032, 0.016, 0.08, 0.4, 2, 10, 50, and 100 *μ*M) for 24 hours. ICA and ICA II were dissolved in dimethyl sulfoxide (DMSO) firstly at concentrations of 20 mM. And it was diluted successively to prepare ICA and ICA II solutions at different concentrations with the culture medium (DMEM solution with 1% DMSO) before use, which could keep ICA and ICA II to be dissolved completely. And cells in control group were also cultured in the same culture medium (DMEM solution with 1% DMSO), which would not induce apoptosis of cells. MTT solution was prepared as 5 mg/mL in PBS just before use and filtered through a 0.22 *μ*m filter. 10 *μ*L of MTT solution was added to each well. After 4 hours of incubation in CO_2_ incubator, the medium containing MTT was removed by inverting and tapping the plates. The intracellular formazan crystals formed were solubilized with 100 *μ*L dimethyl sulfoxide, and the absorbance of the solution was measured at 492 nm by using a microplate reader (Bio-Rad, California, USA). The cell survival rate was calculated as percentage of MTT inhibition by adopting the following formula:

(4) percentage  of  survival=mean  experimental  absorbancemean  control  absorbance×100%.

## 3. Results and Discussion

### 3.1. Zymogram Analysis of *β*-Glucosidase

**Figure 2:**
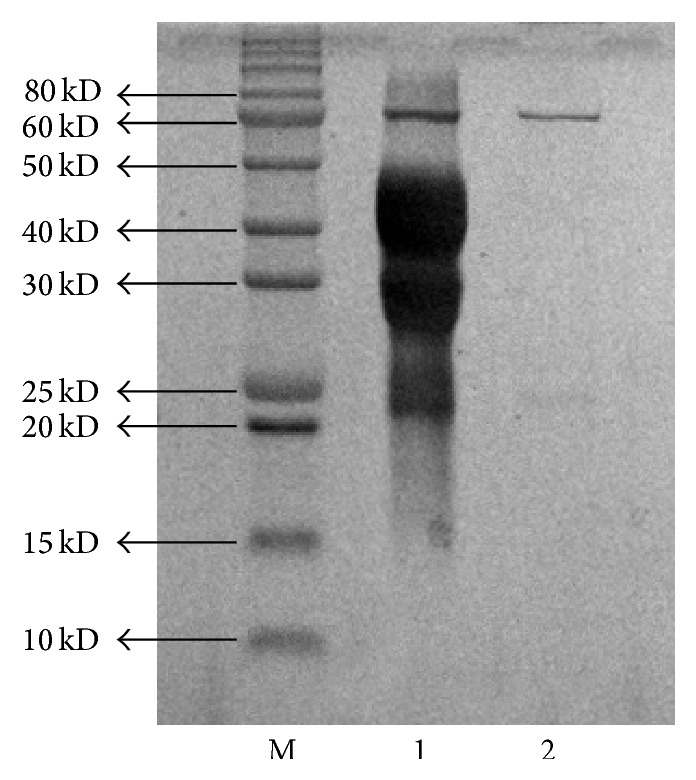
SDS-PAGE of recycled *β*-glucosidase. M: the protein marker; 1: the crude *β*-glucosidase; and 2: the recycled protein from Native-PAGE.

Two kinds of specific substrates of *β*-glucosidase, 4-nitrophenyl-beta-*D*-glucopyranoside (*p*NPG) and 4-methylumbelliferyl-beta-*D*-glucopyranoside (MUG), were adopted to distinguish active bands in Native-PAGE gel [28–30]. These substrates have distinctive color reactions after hydrolysis by *β*-glucosidase. The yellow color characteristic of* p*NPG and fluorescence emission of MU under UV light were observed at the same location of *β*-glucosidase in gel. The target bands were cut down and the protein was retrieved from Native-PAGE gel. The recycled protein was analyzed by SDS-PAGE. There was only one band considered as *β*-glucosidase. Compared with protein marker, the molecular weight of *β*-glucosidase was estimated at 60 kD (Figure 2), which was similar to Mateos et al.'s research [31].

### 3.2. Method Validation

**Figure 3:**
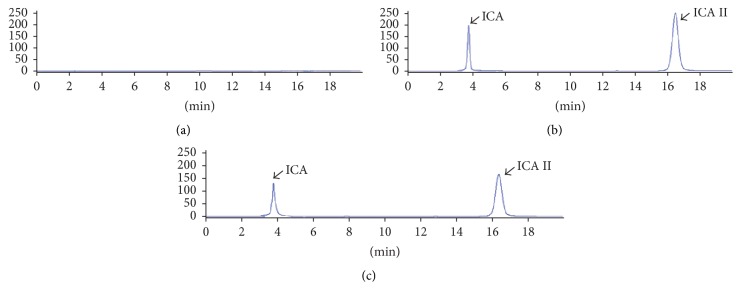
Chromatograms for ICA and ICA II in (a) blank hydrolysis liquid without ICA and ICA II; (b) a standard working solution sample consisting of ICA or ICA II; and (c) a real sample after hydrolysis of ICA.

Under the optimum condition of chromatogram method, there were not any endogenous peaks at retention time of ICA and ICA II, indicating no significant endogenous interference in HPLC analysis method for ICA and ICA II during the process of quantification (Figure 3). The limit of quantification of ICA II was in the range of 4.94–494 *μ*g/mL. The mean linear regression equation for the calibration curve of ICA II was *Y* = 29081*X* − 23.096 (*R* ^2^ = 1, *n* = 3). The calculated LLOQ of ICA II was 1.14 *μ*g/mL (RSD% = 3.5%, *n* = 3). Intra- and interday precisions and accuracies at three concentrations of ICA II ranged from 0.57% to 2.50%. The extraction recoveries of ICA II at three concentrations were all above 95.2%. The stability of ICA II with RSD of 1.67% indicated that ICA II was stable for 12 h in the processed sample after enzymatic hydrolysis. The method validation results showed that this method could be used to quantify ICA II in hydrolysis samples and applied for the enzymatic hydrolysis study of ICA by using *β*-glucosidase.

### 3.3. Monofactor Experiments

**Figure 4:**
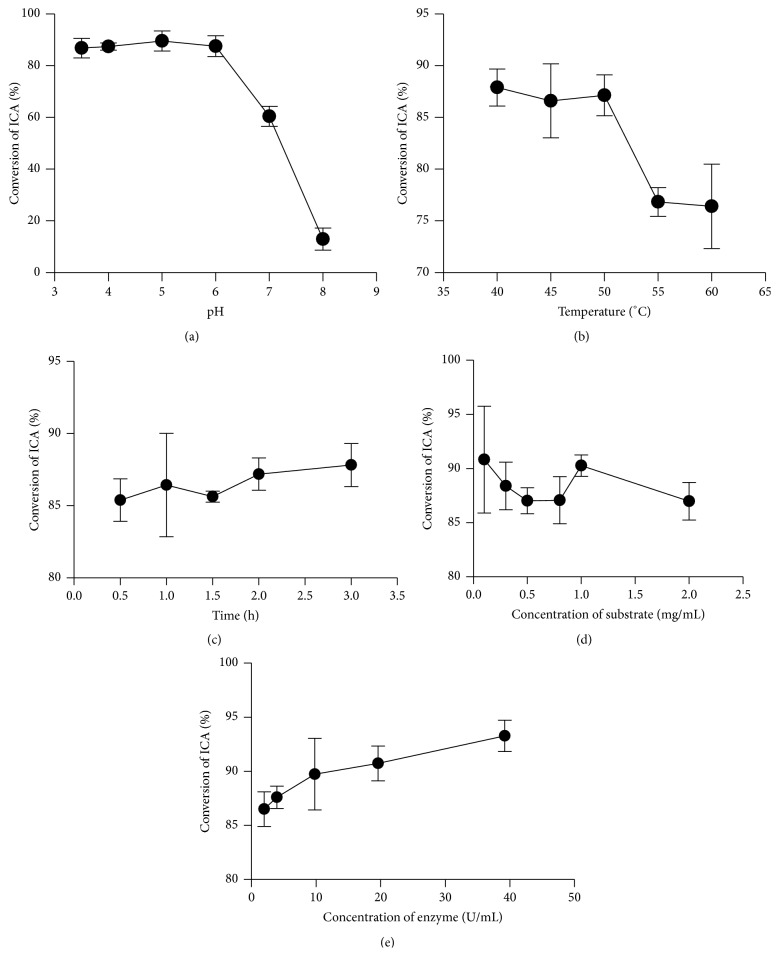
The results of monofactor experiments. (a) The reactions were carried out in sodium acetate-acetic acid buffer (pH 3.5, 4.0, 5.0, 6.0, 7.0, and 8.0) containing ICA (1 mg/mL) and enzyme (19.6 U/mL) at 40°C for 1 h. (b) The reactions were carried out in sodium acetate-acetic acid buffer (pH 5.0) containing ICA (1 mg/mL) and enzyme (19.6 U/mL) at 40°C for 0.5, 1, 1.5, 2, and 3 h. (c) The reactions were carried out in sodium acetate-acetic acid buffer (pH 5.0) containing ICA (1 mg/mL) and enzyme (19.6 U/mL) at 40, 45, 50, 55, and 60°C for 1 h. (d) The reactions were carried out in sodium acetate-acetic acid buffer (pH 5.0) containing ICA (1 mg/mL) and enzyme (1.96, 3.92, 9.8, 19.6, and 39.2 U/mL) at 40°C for 1 h. (e) The reactions were carried out in sodium acetate-acetic acid buffer (pH 5.0) containing ICA (0.1, 0.3, 0.5, 0.8, 1, and 2 mg/mL) enzyme (19.6 U/mL) at 40°C for 1 h.

Reaction time, temperature, pH of buffer, concentration of enzyme, and concentration of substrate were investigated in enzymatic hydrolysis of ICA into ICA II. The conversion rate of ICA decreased significantly from 86.80% to 12.94% when pH changed from 6.0 to 8.0 (Figure 4(a)). Higher conversion rate was achieved in acidic environment. The conversion rate was up to 85.39% after reaction for 0.5 h (Figure 4(b)). The highest conversion rate reached 87.82% after 3 h; however, the increase of the conversion rate was not significant with the extension of the reaction time. So an hour was set as the reaction time for the consideration of efficiency. When the temperature changed from 40°C to 60°C, the conversion rate increased correspondingly from 76.41% to 87.89% (Figure 4(c)). The amount of the enzyme changed from 0.1 mg/mL to 2 mg/mL; the conversion rate of ICA increased from 86.50% to 93.28% (Figure 4(d)). The concentration of substrate was another significant factor affecting the conversion rate. When the substrate concentration changed from 0.1 mg/mL to 0.8 mg/mL, the conversion rate declined from 90.82% to 87.07%. But the conversion rate also declined when the substrate concentration increased from 1 mg/mL to 2 mg/mL (Figure 4(e)), which may be attributed to a poor solubility of ICA and ICA II in water. So the results indicated that pH, temperature, and initial concentration of substrate were the critical factors concerning the conversion of ICA.

### 3.4. Optimization of the Enzymatic Hydrolysis Parameters of ICA by CCD

The choice of the independent variables in CCD experiment was on the basis of results of monoexperiments. pH, temperature, and initial concentration of substrate were the critical factors concerning the conversion of ICA in monoexperiments. The conversion rate of ICA in a faintly acid system was higher than that in an alkalescent system. Temperature was another critical factor as the inherent nature of *β*-glucosidase as a kind of protein that was prone to inactivation in high temperature. ICA was insoluble in aqueous phase which was the medium of this reaction, so the increase of substrate concentration could not enhance the conversion of ICA and ICA could not be homogeneously dispersed in the reaction system instead. Therefore, pH, temperature, and concentration of substrate were chosen to be optimized in the enzymatic hydrolysis of ICA. The result of 20-run CCD in three variables was shown in Table 2.

Regression equation conversion rate of ICA was a function of test variables in coded unit to generate the second-order regression models. The equation is shown as follows:

(5) Y=93.23−6.46X1−0.15X2−1.21X3−1.88X1X2−2.34X1X3−0.60X2X3−4.57X12−4.49X22+0.97X32.

This equation can be used to predict the conversion rate of ICA. The quality of fit of the second-order regression equations was checked by using the coefficient of determination (*R* ^2^) and calculated to be 0.963. The result of the quadratic models for conversion rate of ICA in form of ANOVA was shown in Table 3.

The effects of the variables and their probably existed interactions were analyzed as well. The significance of each coefficient was determined by *p* value. The more significant the corresponding coefficient is, the smaller the *p* value will be. The *p* value of the model was 0.0001 indicating that the model was statistically valid. The pH had a strong positive linear effect on the response (*p* < 0.0001).

**Figure 5:**
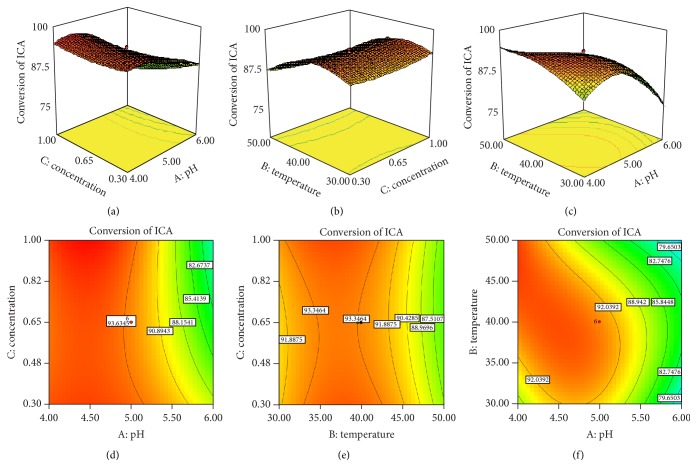
The 2D contour plot and 3D response surface on conversion rate and three influential factors. (a) Temperature and pH at concentration of ICA equal to 0.65 mg/mL; (b) pH and concentration of ICA at a temperature equal to 40°C; (c) temperature and concentration of ICA at a pH equal to 4.0; (d) contour plot for temperature and pH; (e) contour plot for pH and concentration of ICA; and (f) contour plot for temperature and concentration of ICA.

The 3D response surface and 2D contour plot are generally the graphical representation of the regression equation (Figure 5), which depict the interaction between two variables by keeping the other variables at their zero level for conversion rate of ICA. In Table 3, only pH was the crucial element that has a dominant effect on conversion of ICA, which consisted with results of monoexperiments (Figure 4(a)). The range of conversion rate of ICA decreased from 86.80% to 12.94% when pH changed from 6.0 to 8.0. However, conversion rate of ICA at different temperature (20–60°C) and concentration of substrate (0.1–2 mg/mL) approximated a high value with little change from 76.41% to 87.89% (Figure 4(b)) and 90.82% to 87.07% (Figure 4(d)), respectively, which resulted in temperature and concentration of substrate with no significance in the CCD model. However, the optimum pH, temperature, and concentration of substrate selected by the model still can offer a good prediction. In selected optimum conditions of pH 4.0, 41°C, 1 mg/mL ICA in initial reaction system, 9.8 U/mL enzyme, and reaction time of 1 h, the prediction of conversion rate of ICA was 97.28% and the actual one was up to 95.03%, with the deviation of the test at 2.31%, which resulted from a large amount of repetitive tests. Subsequently, the optimized condition selected by the model in this paper was applied to enlarged production of ICA II, which had gained a high and stable conversion rate of ICA, and achieved the purpose of mass preparation of ICA II by biotransformation of ICA. It was not the first report in which statistic method was applied to optimize the production of ICA II. Park et al. [32] also adopted CCD method and optimum production conditions were enzyme concentration of 7.5 mg/mL, pH 5, 50°C, and reaction time of 12 h. The yield was 94.14%. Xia et al. [33] used an orthogonal array design (OAD) experiment (L_18_(34)) method and the optimized reaction conditions were 50°C, pH 6.0, and reaction time of 5 h and the conversion rate of ICA was 62.80%. Yang et al. [34] used response surface and subset selection method and the optimized reaction at room temperature (nearly 22°C), pH 5.4, and a reaction time of 3 h, which resulted in a conversion rate of 93.8%. In this paper, the reaction time was apparently shorter with a satisfactory conversion rate.

### 3.5. Identification of the Product

The product purity reached 98.26% after recrystallization. The analysis results were as follows: IR: 3231.52, 2926.71, 1654.74, 1608.87, 1571.54, 1428.03, 1364.27, 835.05; UVnm: (log⁡*ε*): 205 (sh, 2.04), 270 (1.63), 300 (0.86), 350 (0.67); NMR: ^1^H-NMR (DMSO-*d*_6_, 400 MHZ) *δ* 6.32 (1H, s, H-6), 3.50 (2H, m, H-11, overlapped), 5.15 (1H, t, *J* = 6.0 Hz, H-12), 1.62 (3H, s, H-14), 1.68 (3H, s, H-15), 12.52 (1H, s, 5-OH), 7.86 (2H, d, *J* = 8.8 Hz, H-2′, 6′), 7.12 (2H, d, *J* = 8.7 Hz, H-3′, 5′), 3.84 (3H, s, 4′-OCH_3_), 5.26 (1H, br s, H-1_3-O-Rha_), 0.77 (3H, d, *J* = 5.9 Hz, H-6-Rha); ^13^C-NMR (DMSO-*d*_6_, 100 MHz): *δ* 156.72 (C-2), 134.42 (C-3), 177.97 (C-4), 161.26 (C-5), 98.32 (C-6), 161.61 (C-7), 105.92 (C-8), 153.76 (C-9), 104.16 (C-10), 21.14 (C-11), 122.39 (C-12), 131 (C-13), 25.39 (C-14), 17.76 (C-15), 122.25 (C-1′), 130.38 (C-2′), 114.03 (C-3′), 158.83 (C-4′), 114.03 (C-5′), 130.38 (C-6′), 55.46 (4′-OMe), 101.95 (C-1′′_3-O-Rha_), 70.05 (C-2′′), 70.30 (C-3′′), 71.10 (C-4′′), 70.62 (C-5′′), 17.43 (C-6′′). The structure was confirmed by comparison of its UV, IR, ESI-MS, ^1^H-NMR, and ^13^C-NMR spectra with [32]. The ^1^H-NMR and ^13^C-NMR spectra are shown in Figure S1 of the Supplementary Material available online at http://dx.doi.org/10.1155/2016/5936947.

### 3.6. Cytotoxicity of ICA and ICA II on Human Lung Adenoma A549 Cells

**Figure 6:**
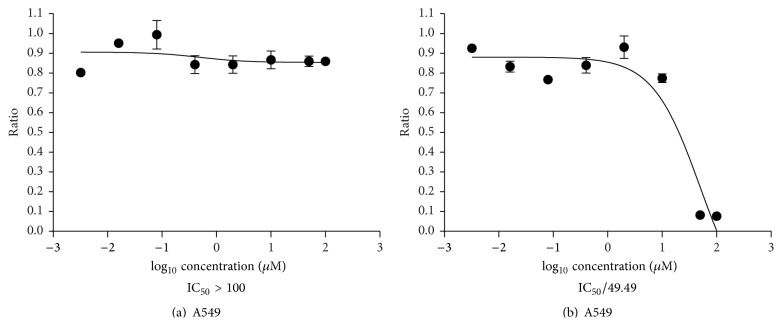
Graphical representation of MTT assay to determine IC_50_ value of ICA (a) and ICA II (b).

The cell viability of A549 cells was examined after treatment of ICA and ICA II at 24 hours. A dose-dependent cytotoxicity was obtained in Figure 6, which showed that ICA has no effects on proliferation of A549 cell. But its metabolite, ICA II, performed differently and its IC_50_ value was found to be 49.49 *μ*M. The results indicated that the hydrolysis reaction of ICA is valuable for exploration of more active new drug.

## 4. Conclusions

A specific *β*-glucosidase produced from* Trichoderma viride* was used to biotransform ICA into ICA II. The Mol. wt. of the *β*-glucosidase was revealed at 60 kD by SDS-PAGE analysis. Through a monoexperiment and CCD experiments, it is ascertained that the optimum parameters of biotransformation of ICA were pH at 4.0, temperature of 41°C, 1.0 mg/mL ICA, and 9.8 U/mL crude *β*-glucosidase, and at that time the conversion rate of ICA reached 95.03% in 1 h. The process costs less time and acquires a higher hydrolysis rate of ICA compared with other researches. Moreover, ICA II performed inhibition effects on proliferation of A549 cell, while ICA has no cytotoxicity, which predicted that ICA II may be a potential new drug with high activities.

## Supplementary Material

The NMR Spectrum of ICA II :^1^H-NMR(DMSO-d_6_, 400 MHZ) δ 6.32 (1H, s,H-6), 3.50 (2H, m, H-11, overlapped), 5.15 (1H, t, J = 6.0 Hz, H-12), 1.62 (3H, s, H-14), 1.68 (3H, s, H-15), 12.52 (1H, s, 5-OH), 7.86 (2H, d, J = 8.8 Hz, H-2′, 6′), 7.12 (2H, d, J = 8.7 Hz, H-3′, 5′), 3.84 (3H, s, 4′-OCH_3_), 5.26 (1H, br s, H-1_3-O-Rha_), 0.77 (3H, d, J = 5.9 Hz, H-6-Rha); ^13^C-NMR(DMSO-d_6_, 100 MHz): δ 156.72 (C-2), 134.42 (C-3), 177.97(C-4), 161.26 (C-5), 98.32 (C-6), 161.61 (C-7), 105.92 (C-8), 153.76 (C-9), 104.16 (C-10), 21.14 (C-11), 122.39 (C-12), 131 (C-13), 25.39 (C-14), 17.76 (C-15), 122.25 (C-1′), 130.38 (C-2′), 114.03 (C-3′), 158.83 (C-4′), 114.03 (C-5′), 130.38 (C-6′), 55.46 (4′-OMe), 101.95 (C-1"_3-O-Rha_), 70.05(C-2"), 70.30 (C-3"), 71.10 (C-4"), 70.62 (C-5"), 17.43 (C-6").

## Figures and Tables

**Table 1:** Levels of variables used in the experimental design.

Independent variables	Levels				
	−*α*	−1	0	1	*α*
pH (*X*_1_)	3.32	4.0	5.0	6.0	6.68
Temperature (*X*_2_) (°C)	23.18	30	40	50	56.86
Concentration of substrate (*X*_3_) (mg mL^−1^)	0.30	0.30	0.65	1.00	1.24

*α*: 1.682.

**Table 2:** Experimental design with real value and predicted value of conversion rate of ICA.

Run	*X*_1_ (pH)	*X*_2_ (temperature, °C)	*X*_3_ (concentration of substrate, mg/mL)	*Y* (conversion rate of ICA, %)	
				Experimental	Predicted
1	4.0	30	0.30	89.26	88.14
2	5.0	40	0.65	93.90	93.23
3	5.0	40	0.65	92.99	93.23
4	5.0	40	1.24	95.32	93.92
5	3.3	40	0.65	88.40	91.15
6	6.0	30	1.00	75.38	77.75
7	6.0	50	0.30	83.67	80.79
8	4.0	50	0.30	96.15	92.78
9	4.0	30	1.00	89.71	91.59
10	5.0	57	0.65	75.44	80.28
11	4.0	50	1.00	96.67	93.85
12	5.0	40	0.65	92.43	93.23
13	6.7	40	0.65	70.76	69.43
14	5.0	40	0.65	93.04	93.23
15	6.0	30	0.30	81.84	83.66
16	6.0	50	1.00	72.37	72.49
17	5.0	23	0.65	84.21	80.79
18	5.0	40	0.06	95.18	98.00
19	5.0	40	0.65	94.07	93.23
20	5.0	40	0.65	93.17	93.23

**Table 3:** ANOVA of the quadratic regression model for conversion rate of ICA.

Source	SS	MS	DF	*F*	Prob. > *F*
Model	1249.94	138.88	9	14.24	0.0001
*X*_1_	569.58	569.58	1	58.40	<0.0001
*X*_2_	0.32	0.32	1	0.032	0.8606
*X*_3_	20.07	20.07	1	2.06	0.1820
*X*_1_ *X*_2_	28.24	28.24	1	2.90	0.1197
*X*_1_ *X*_3_	43.85	43.85	1	4.50	0.0600
*X*_2_ *X*_3_	2.84	2.84	1	0.29	0.6010
*X*_1_^2^	301.53	301.53	1	30.91	0.0002
*X*_2_^2^	290.22	290.22	1	29.76	0.0003
*X*_3_^2^	13.45	13.45	1	1.38	0.2675
Residual	97.54	9.75	10		
Lack of fit	95.65	19.13	5	50.78	0.0003
Pure error	1.88	0.38	5		
Total	1347.48		19		
