# Supplementary figures and images for: Optimized Biotransformation of Icariin into Icariside II by β-Glucosidase from Trichoderma viride Using Central Composite Design Method
Source: Biomed Res Int. 2016 Feb 14;2016:5936947. doi: 10.1155/2016/5936947 (PMC4769847; doi:10.1155/2016/5936947)

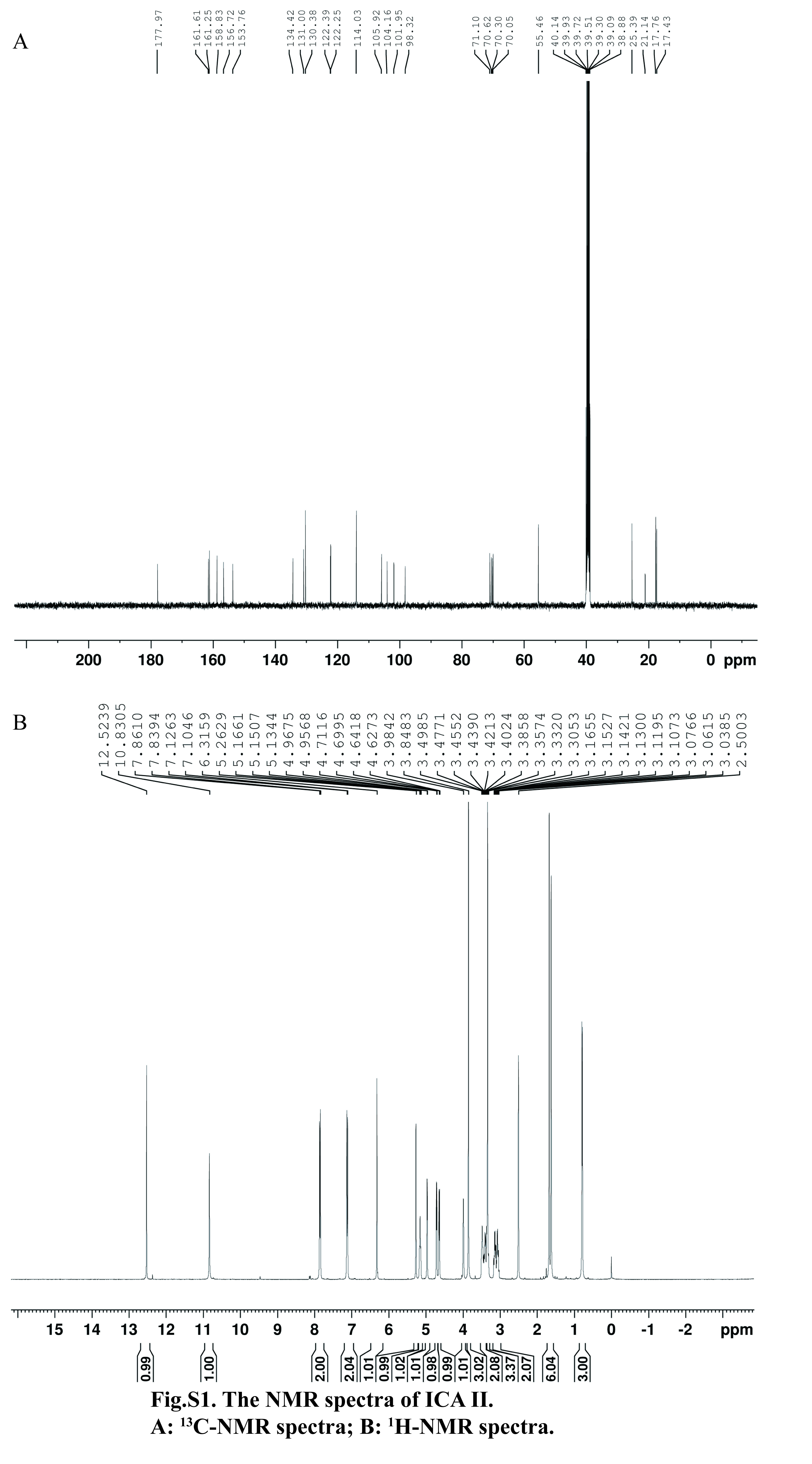

Supplement: Supplementary file 1 — The NMR Spectrum of ICA II :1H-NMR(DMSO-d6, 400 MHZ) δ 6.32 (1H, s,H-6), 3.50 (2H, m, H-11, overlapped), 5.15 (1H, t, J = 6.0 Hz, H-12), 1.62 (3H, s, H-14), 1.68 (3H, s, H-15), 12.52 (1H, s, 5-OH), 7.86 (2H, d, J = 8.8 Hz, H-2′, 6′), 7.12 (2H, d, J = 8.7 Hz, H-3′, 5′), 3.84 (3H, s, 4′-OCH3), 5.26 (1H, br s, H-13-O-Rha), 0.77 (3H, d, J = 5.9 Hz, H-6-Rha); 13C-NMR(DMSO-d6, 100 MHz): δ 156.72 (C-2), 134.42 (C-3), 177.97(C-4), 161.26 (C-5), 98.32 (C-6), 161.61 (C-7), 105.92 (C-8), 153.76 (C-9), 104.16 (C-10), 21.14 (C-11), 122.39 (C-12), 131 (C-13), 25.39 (C-14), 17.76 (C-15), 122.25 (C-1′), 130.38 (C-2′), 114.03 (C-3′), 158.83 (C-4′), 114.03 (C-5′), 130.38 (C-6′), 55.46 (4′-OMe), 101.95 (C-1"3-O-Rha), 70.05(C-2"), 70.30 (C-3"), 71.10 (C-4"), 70.62 (C-5"), 17.43 (C-6"). [file 5936947.f1.jpg]
